# Supplementary material for: Abundance-weighted phylogenetic diversity measures distinguish microbial community states and are robust to sampling depth
Source: PeerJ. 2013 Sep 12;1:e157. doi: 10.7717/peerj.157 (PMC3775626; doi:10.7717/peerj.157)
Supplement: Supplemental Information 1 — PDF containing a derivation and a more general description of weighted PD, as well as a number of supplementary figures and tables. [file peerj-01-157-s001.pdf]

1. Appendix

1.1. **Rarefaction of phylogenetic quadratic entropy.** We investigate the rarefaction of phylogenetic quadratic entropy (PQE), which is a diversity coefficient in the language of (Rao, 1982) and defined as follows (Warwick and Clarke, 1995; Allen et al., 2009). If the tree is not rooted, root it arbitrarily (the rooting does not impact the value). PQE is defined on a tree as

$$\mathrm{PQE}_k = \sum_i \ell_i \frac{a_i}{n}\left(1 - \frac{a_i}{n}\right), \tag{1}$$

where ℓ_i is the length of edge i and a_i is the number of leaf observations that are distal (away from root) from edge i.

Assume we rarefy to k observations as above; let A_i denote the random variable that is the number of observations distal to edge i after rarefaction. The phylogenetic quadratic entropy is then

$$\mathrm{PQE}_k = \sum_i \ell_i \frac{A_i}{k}\left(1 - \frac{A_i}{k}\right). \tag{2}$$

The random variable A_i has a hypergeometric distribution, performing k draws with d_i possible successes in a population of size n. Let μ_i be the expectation of A_i, which is simply kd_i/n. The variance of the hypergeometric distribution is well known to be

$$\sigma_i^2 = \frac{kd_i(n-d_i)(n-k)}{n^2(n-1)}. \tag{3}$$

Next

$$\begin{aligned}\mathbb{E}[\mathrm{PQE}_k] &= \sum_i \ell_i \mathbb{E}\left[\frac{A_i}{k}\left(1 - \frac{A_i}{k}\right)\right], \\ &= \frac{1}{k^2}\sum_i \ell_i \left(k\mathbb{E}[A_i] - \mathbb{E}[A_i^2]\right).\end{aligned}$$

By definition,

$$\mathbb{E}[A_i^2] = \mu_i^2 + \sigma_i^2.$$

Thus the expectation of the phylogenetic quadratic entropy upon rarefaction is

$$\mathbb{E}[\mathrm{PQE}_k] = \frac{1}{k^2}\sum_i \ell_i (k\mu_i - \mu_i^2 - \sigma_i^2). \tag{4}$$

Expanding the term in parentheses from (4):

$$\begin{aligned}k\mu_i - \mu_i^2 - \sigma_i^2 &= k^2\frac{d_i}{n} - k^2\frac{d_i^2}{n^2} - \frac{kd_i(n-d_i)(n-k)}{n^2(n-1)} \\ &= kd_i\frac{kn(n-1) - kd_i(n-1) - (n-d_i)(n-k)}{n^2(n-1)} \\ &= kd_i\frac{(n-d_i)(k(n-1) - (n-k))}{n^2(n-1)} \\ &= \frac{k(k-1)}{n(n-1)}d_i(n-d_i)\end{aligned}$$

Putting this back in (4), we obtain

$$\mathbb{E}[\mathrm{PQE}_k] = \frac{k-1}{kn(n-1)} \sum_i \ell_i d_i (n - d_i) \tag{5}$$

In principle one could calculate the variance of phylogenetic quadratic entropy in terms of the higher order moments of the hypergeometric distribution. However, these higher moments are very messy and we have not attempted to write out the variance calculation. We also note that this derivation could be easily generalized to the setting of a "tree with marks" as in (Nipperess and Matsen, 2012).

1.2. **Description of PD in more general setting.** We can describe the methods in the general setting where samples are represented by a mass distribution on a tree. As described elsewhere (Evans and Matsen, 2012), this generalizes the notion of representing a sample by an OTU count equipped with a phylogenetic tree on OTU representative. Specifically, if the total sample size is N, then n observations of a given OTU ω are represented by a point mass of weight n/N at ω.

As observed by others (Allen et al., 2009) phylogenetic diversity measures can be written as

$$\mathrm{PD_u}(s) = \sum_i \ell_i F(D_s(i)) \tag{6}$$

where F is some real-valued function on the unit interval. This can be further generalized to the case of an abitrary probability distribution by writing this as an integral where λ is the length measure on the tree (Evans and Matsen, 2012) and now $D_s(y)$ is the total mass on the distal side of y.

$$\mathrm{PD_u}(s) = \int_{y \in T} F(D_s(y))\, \lambda(dy) \tag{7}$$

For phylogenetic quadratic diversity, $F(x) = x(1-x)$, and for phylogenetic entropy, $F(x) = -x \log x$. As described above, the BWPD_θ fits into this framework with $F(x) = \min(g_\theta(x), g_\theta(1-x))$.

1.3. **Analysis of oral dataset with additional quality filtering steps.** After the quality filtering steps reported in the main text, sequences were error-corrected using Acacia (Bragg et al., 2012), and putative chimeras identified by UCHIME (Edgar et al., 2011) were removed. Results are shown in Table S1.

References

B. Allen, M. Kon, and Y. Bar-Yam. A new phylogenetic diversity measure generalizing the Shannon index and its application to phyllostomid bats. *The American Naturalist*, 174(2):236–243, 2009.

L. Bragg, G. Stone, M. Imelfort, P. Hugenholtz, and G.W. Tyson. Fast, accurate error-correction of amplicon pyrosequences using acacia. *Nature Methods*, 9(5): 425–426, 2012.

R.C. Edgar, B.J. Haas, J.C. Clemente, C. Quince, and R. Knight. UCHIME improves sensitivity and speed of chimera detection. *Bioinformatics*, 27(16):2194–2200, 2011.

S.N. Evans and F.A. Matsen. The phylogenetic Kantorovich-Rubinstein metric for environmental sequence samples. *Journal of the Royal Statistical Society: Series B*, 74(3):569–592, 2012.

D.A. Nipperess and F.A. Matsen. The mean and variance of phylogenetic diversity under rarefaction. *Submitted to Methods in Ecology and Evolution*, 2012. arXiv:1208.6552.

Julia Oh, Sean Conlan, E Polley, Julia A Segre, Heidi H Kong, et al. Shifts in human skin and nares microbiota of healthy children and adults. *Genome medicine*, 4 (10):1–11, 2012.

C.R. Rao. Diversity and dissimilarity coefficients: a unified approach. *Theoretical Population Biology*, 21(1):24–43, 1982.

R.M. Warwick and K.R. Clarke. New 'biodiversity' measures reveal a decrease in taxonomic distinctness with increasing stress. *Marine Ecology Progress Series*, 129(1):301–305, 1995.

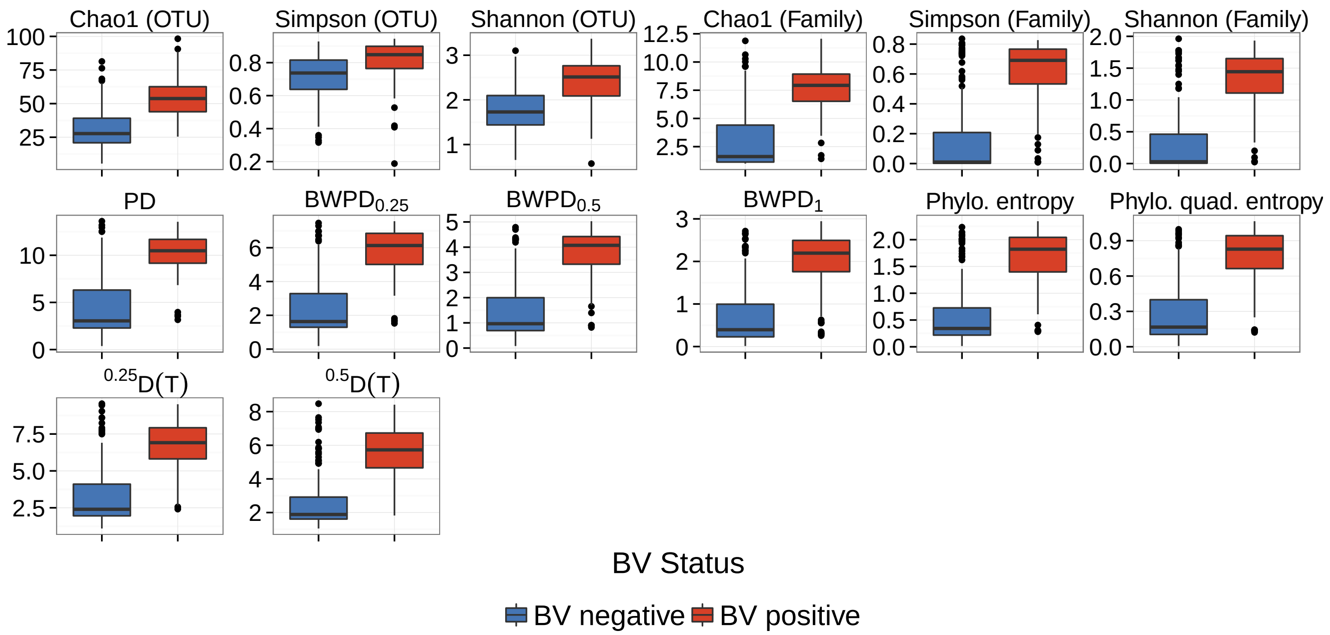

FIGURE S1. Comparison of diversity between samples from BV negative and BV positive women, using different measures of alpha diversity. Top row: cluster-based methods. Bottom rows: phylogenetic methods.

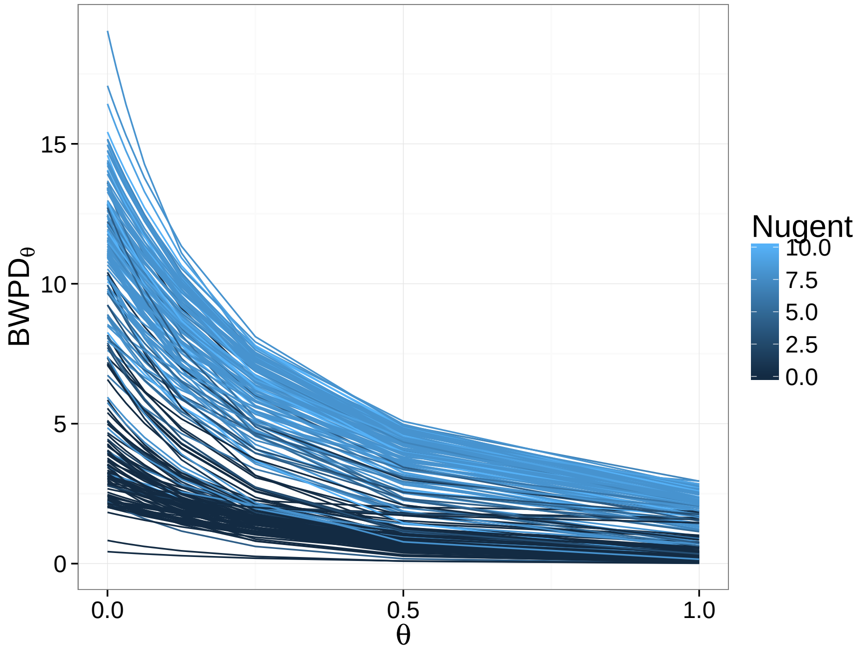

FIGURE S2. Values of $BWPD_\theta$ for various θ. Each line represents a specimen from the vaginal dataset. Lines are colored by Nugent score. A Nugent score of 7–10 is consistent with bacterial vaginosis.

Measure	Diseased status accuracy	ANOVA p-value	mean rank
Phylo. entropy	0.798	3.43E-09	1.0
$BWPD_{0.5}$	0.793	4.18E-09	2.0
$BWPD_{0.25}$	0.784	3.22E-08	3.5
Simpson (Family)	0.791	1.09E-06	4.0
Phylo. quad. entropy	0.770	2.21E-07	4.5
PD_u	0.724	1.47E-06	7.0
${}^{0.5}D(T)$	0.736	2.39E-06	7.0
Shannon (Family)	0.766	2.71E-05	7.5
${}^{0.25}D(T)$	0.711	1.38E-05	8.5
$BWPD_1$	0.699	3.01E-04	11.0
Chao1 (OTU)	0.705	6.78E-04	11.0
Shannon (OTU)	0.688	2.83E-04	11.5
ACE (OTU)	0.693	1.13E-03	12.5
Simpson (OTU)	0.674	2.67E-02	14.5
Chao1 (Family)	0.674	1.62E-01	15.0
ACE (Family)	0.663	1.09E-01	15.5

TABLE S1. Predictive accuracy of each measure in the oral dataset and p-value from an ANOVA stratified by disease status and sampling site after additional quality filtering.

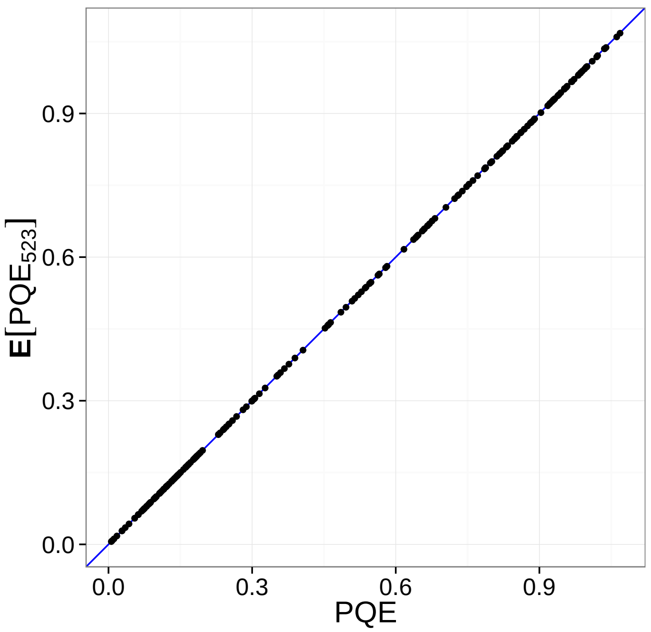

Figure S3. Comparison of phylogenetic quadratic entropy (PQE) calculated on all sequences from the vaginal dataset to the expectation of PQE expectation under rarefaction to 523 sequences per specimen (the smallest sequence count across all specimens) computed via our analytical formula. The $y = x$ line is shown in blue.

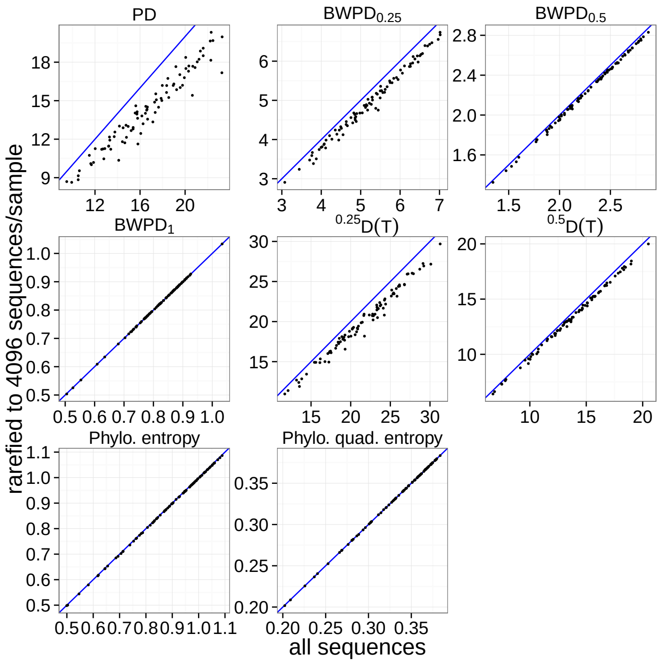

FIGURE S4. Comparison of rarefied and unrarefied values of various phylogenetic alpha diversity measures as applied to the oral dataset. The value of six alpha measures for each specimen using all available sequences is plotted on the x-axis. The value of the alpha measure for each specimen after a single rarefaction to 4,096 sequences (the smallest sequence count across specimens) is plotted on the y-axis. The $y = x$ line is shown in blue.

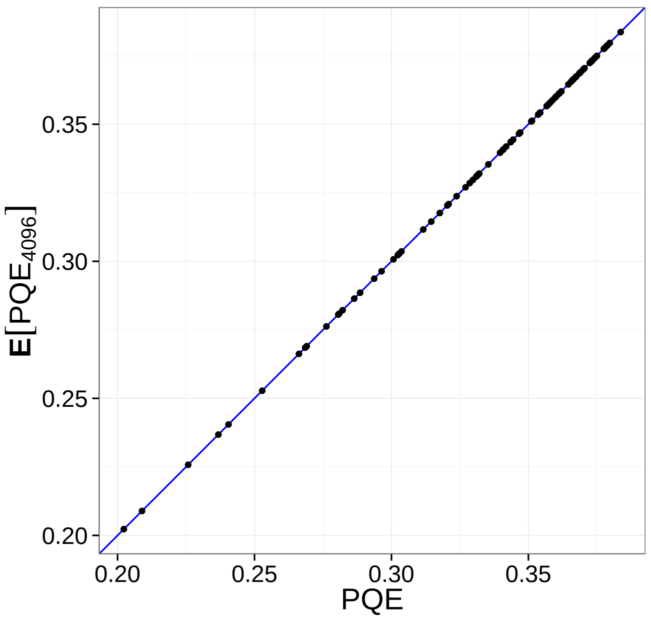

FIGURE S5. Comparison of phylogenetic quadratic entropy (PQE) calculated on all sequences from the oral dataset to the analytically-derived expectation of PQE under rarefaction to 4096 sequences per specimen (the smallest sequence count across all specimens) computed via our analytical formula. The $y = x$ line is shown in blue.

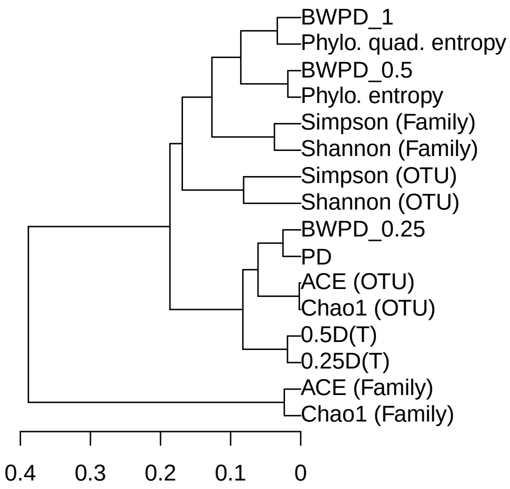

Figure S6. Dendrogram relating alpha diversity measures applied to the oral dataset.

	Ac	N	Pc	Vf	mean rank
PD_u	2.28e-02	6.35e-03	2.33e-03	1.41e-04	4.50
$BWPD_{0.25}$	2.88e-02	1.27e-03	5.30e-03	4.72e-04	5.00
$BWPD_{0.5}$	6.11e-02	6.59e-05	3.72e-02	5.92e-03	5.50
Chao1 (OTU)	4.84e-02	6.98e-03	2.97e-03	6.37e-03	6.50
Shannon (OTU)	6.32e-02	8.26e-02	7.82e-02	1.39e-05	7.00
Phylo. quad. entropy	2.11e-01	5.81e-06	5.63e-01	1.76e-01	7.50
Phylo. entropy	9.55e-02	6.28e-04	1.71e-01	2.02e-02	7.75
$^{0}D(T)$	3.09e-01	6.13e-03	1.63e-03	9.51e-01	8.25
$BWPD_1$	2.65e-01	2.68e-05	7.25e-01	5.61e-01	8.50
$^{0.5}D(T)$	8.14e-01	4.10e-04	7.57e-03	9.30e-01	8.75
Simpson (OTU)	8.81e-02	3.56e-01	8.39e-01	1.04e-04	8.75
$^{0.25}D(T)$	5.57e-01	1.42e-03	3.47e-03	9.56e-01	9.25

Table S2. ANOVA p-values for various phylogenetic diversity statistics applied to the skin microbiome data of Oh et al. (2012). Rows are ordered by increasing mean rank across sites. The same site abbreviations are used as in their paper: Ac, antecubital fossa; N, nares; Pc, popliteal fossa; Vf, volar forearm. Putative chimeras identified by UCHIME (Edgar et al., 2011) were removed prior to analysis.
